# Practical Notes on Diagnosis and Treatment

**Published:** 1910-02-26

**Authors:** 


### Practical Notes on Diagnosis and Treatment.

**To Abort Boils.**

A boil can often be aborted by covering it with three or four layers of tincture of iodine.—*Prof. Robin.*

**Tea in Gout.**

The value of tea, taken weak and freshly made, is very great as an aid in eliminating uric acid. Green vegetables should be freely partaken of, but sweet fruits avoided.—*Mr. J. Hutchinson.*

**Insect Bites.**

The pain from the bites of mosquitoes and the stings of wasps and bees may be quickly relieved by rubbing on a few drops of petroleum containing iodine 30 to 40 gr. to the ounce.

**Imperial Drink.**

This is often made up as follows: Citric acid, gr. xv. (or the juice of half a lemon); acid potassium tartrate, gr. 60; sugar, 1 oz.; water, 1 pint. More sugar is often preferred and tartaric acid, gr. 60, with about 2m. of oleum limonis may replace the citric acid.

**Thrush.**

This affection should be regarded as an indication for combating a state of exhaustion into which the patient has fallen. Hence stimulants and quinine and strychnine are useful. When the health is restored the thrush will disappear, but to expedite matters the mouth may be cleansed with warm bicarbonate of soda solution (5 grains to the ounce).

**Delirium Tremens.**

If you intend to give opium, first make sure that the kidneys are sound, then give it boldly. Begin with 60m. of laudanum, rather than with 20 and then 40 and then 60; the latter method is not only ineffectual but often dangerous, and may induce a tolerance which will defeat your object. Every effort must be made to get nourishment into the patient; until his nutrition improves progress will be slow and opiates likely to fail.

**Cough.**

The causes of cough are exceedingly numerous, but it is useful to remember that a cough which wakes the patient up after he has been asleep is probably due to lung disease.

**Cough Medicines.**

Remember that nearly all cough mixtures tend to upset the digestion of children. A simple febrifuge mixture should always be preferred, and will often prove sufficient throughout the illness, unless there is a clear demand to aid or diminish expectoration.

**Pruritus Ani.**

The cause of some obstinate cases of pruritus ani is the presence of a small ulcer on the rectal mucous membrane, between the external and internal sphincters. The best treatment is forcible stretching of the sphincter.

**Vaccine Treatment of Septic Conditions.**

In St. Thomas's the vaccine treatment of septic cases has been distinctly disappointing. There is no appreciable difference in the time spent in the hospital by those who had vaccines and those who had not (for carbuncle). With regard to septicæmia, pyæmia, and septic bone disease, the results have been equally indifferent.—*Mr E. M. Corner.*

**Summer Diarrhœa.**

It is an excellent plan to place the infant face downwards on its mother's lap and give about a pint of water (at 100° F.) per rectum. It may be advisable to give a hypodermic of morphia—1-30 grain for a child 12 months old. Feeding by the mouth in small quantities at frequent intervals should be tried—*e.g.* ʒj. peptonised milk with 1m. of brandy every 15 minutes, or beef-juice, or nothing but albumen water. If there be no vomiting give 1m. Liq. Hydrarg. Perchlor. with ½m. Sp. Chlorof. in one drachm of water every three hours.—*Dr. John Abercrombie.*

